# Assessment of the efficacy of palliative sedation in advanced cancer patients by evaluating discomfort levels: a prospective, international, multicenter observational study

**DOI:** 10.1186/s12916-024-03829-7

**Published:** 2024-12-31

**Authors:** Maaike Rijpstra, Kris Vissers, Alazne Belar, Michael Van der Elst, Séverine Marie Surges, Claudio Adile, Rocío Rojí, Yasmine Grassi, Ewald Bronkhorst, Sebastiano Mercadante, Lukas Radbruch, Johan Menten, Carlos Centeno, Evelien Kuip, Jeroen Hasselaar

**Affiliations:** 1 Department of Primary and Community Care, Radboud University Medical Centre, Radboud Institute for Health Sciences, Geert Grooteplein 10, Nijmegen, 6500HB the Netherlands; 2 Department of Anaesthesiology, Pain and Palliative Medicine, Radboud University Medical Centre, Radboud Institute for Health Sciences, Nijmegen, the Netherlands; 3 IdISNA-Instituto de Investigación Sanitaria de Navarra, Palliative Medicine, Pamplona, Spain; 4 Institute for Culture and Society-ATLANTES, Universidad de Navarra, Pamplona, Navarra Spain; 5 Department of Oncology, Laboratory of Experimental Radiotherapy, Hospital and Catholic University Leuven, Louvain, Belgium; 6 Department of Palliative Medicine, University Hospital Bonn, Bonn, Germany; 7 Main Regional Center of Supportive/Palliative Care, La Maddalena Cancer Center, Palermo, Italy; 8 Department Of Palliative Medicine, Clinica Universidad de Navarra, Pamplona, Spain; 9 Department for Health Evidence, Radboud University Medical Centre, Radboud Institute for Health Sciences, Nijmegen, The Netherlands

**Keywords:** Palliative sedation, Monitoring, Observational study, End-of-life care, Advanced cancer

## Abstract

**Background:**

Palliative sedation involves the intentional proportional lowering of the level of consciousness in patients with life-limiting disease who are experiencing refractory suffering. The efficacy of palliative sedation needs to be monitored to ensure patient comfort. The aim of this study was to evaluate the efficacy using discomfort levels combined with sedation/agitation levels.

**Methods:**

In this prospective observational study, adult patients with advanced malignancies were recruited from hospice units, palliative care units, and hospital wards in five European countries. Health care professionals used proxy observations of discomfort levels (Discomfort Scale-Dementia of Alzheimer Type, range 0–27) and sedation/agitation levels (Richmond Agitation-Sedation Scale modified for palliative care inpatients), range − 5 to + 4) to evaluate the efficacy of palliative sedation.

**Results:**

In 78 participants, discomfort levels were monitored during palliative sedation. The mean discomfort score before start was 9.4 points (95% CI 8.3–10.5), which showed a significant decrease of 6.0 points (95% CI 4.8–7.1) after start of sedation for the total sedation period. In the multivariable analysis, no significant factors influencing baseline discomfort levels were identified. The discomfort and depth of sedation scores were found to be positively correlated, with an *r* of 0.72 (95% CI 0.61–0.82). The internal consistency of the discomfort scale was good (0.83), but the “Noisy breathing” item was less informative of the total discomfort score.

**Conclusions:**

The efficacy of palliative sedation can be evaluated by measuring discomfort levels combined with sedation/agitation levels. The measurement of discomfort levels might provide a more specific and detailed evaluation of adequate sedation**.**

**Trial registration:**

This study is registered at ClinicalTrials.gov since January 22, 2021, registration number: NCT04719702.

## Background

The number of people diagnosed with cancer is increasing every year in European countries [1], resulting in higher number of patients with advanced cancer and, consequently, an increasing need for palliative care [2].

In most patients with advanced cancer, conventional symptom treatment is sufficient to control symptom distress [3]. However, for some patients, treatment options become limited at the end of life, either due to a reduction in the desired effect of a specific treatment, the lack of an acceptable period to reach symptom control, or the presence of too many treatment side effects. Consequently, symptoms can become refractory, causing unbearable suffering for patients who are nearing death [4]. For patients with limited life expectancy and refractory suffering, palliative sedation can be a last resort clinical option [5].

Palliative sedation is the intentional and proportional lowering of the level of consciousness at the end of life to alleviate refractory suffering [6, 7]. To guide this clinical practice, several (inter)national guidelines have been developed [8]. Although the importance of monitoring efficacy is mentioned in most palliative sedation guidelines, evidence on the variables that need to be monitored, how to monitor such variables, and how often monitoring should be performed is ambiguous or lacking [8, 9].

Previous studies predominantly used outcome measures such as depth of sedation to evaluate palliative sedation practice [9]. However, the purpose is not primarily acquiring a deeply sedated patient but offering proportional sedation to reach a state of comfort [7]. Since achieving comfort is the major goal, (dis)comfort seems to be a more appropriate indicator of the efficacy, as has been used by some studies [10]. Additionally, items in (dis)comfort measurement scales are more in line with the variables monitored by healthcare providers in daily clinical practice for the evaluation of palliative sedation [11].

Finally, international, multicenter prospective trials studying clinical pathways to monitor the efficacy of palliative sedation in different palliative care settings are still lacking [4]. Since previous research reported the influence setting-specific aspects on the clinical practice of palliative sedation [12, 13], those variations should be included in the interpretation of the results. Prospective, international multicenter studies could be valuable for implementing clinically relevant outcome measures to accurately evaluate the effects of palliative sedation.

The main objective of this study was to evaluate the efficacy of palliative sedation on patients’ discomfort levels. Second, the depth of sedation was measured, and its correlation with the level of discomfort was explored. Finally, we briefly evaluated the use of a proxy discomfort measurement scale within a sample of patients receiving palliative sedation.

## Methods

### Study design and settings

This study was part of the international European Horizon 2020 (H2020) “Palliative Sedation” project. In this prospective, observational clinical study, participants were followed during their admission to one of the participating palliative care settings. During the study period from April 2021 to April 2023, participants were recruited from palliative care units (PCUs) (Belgium, *n* = 1; Germany, *n* = 2), hospice units (Italy, *n* = 1; the Netherlands, *n* = 2), and oncology wards of hospitals with a consulting palliative care team (Spain, *n* = 1; the Netherlands, *n* = 2).

### Study population

Adult advanced cancer patients with confirmed or possible development of refractory suffering, a limited life expectancy, and the ability to provide informed consent were eligible for inclusion. The timing of inclusion was dependent on local practices as well as the patient’s ability to provide informed consent. As a result, the timing of inclusion varied regarding the patient’s trajectory, as did the life expectancy (≤ 3 or ≤ 6 months). Discharged and readmitted participants were asked whether they still agreed to participate in the study.

Baseline measurements were completed at inclusion or at the start of the second admission. Then, the follow-up period started, and study measurements were continued for participants for whom palliative sedation was initiated.

Within this study, the following definition for palliative sedation, based on the 2009 European Association for Palliative Care (EAPC) framework, was used [6]: “the monitored use of medications intended to induce a state of decreased or absent awareness in order to relieve the burden of otherwise intractable suffering.” During the decision-making process, the attending health care professionals (HCPs) indicated that the aim for starting sedative medication was palliative sedation. After confirmation of the aim, monitoring of the process started. The participating settings followed the national or local guidelines for palliative sedation. The leading principle in sedation was proportionality to the need for the relief of suffering. Therefore, different forms (intermittent or continuous) and depths of sedation (light to deep) were allowed for participants in the study.

### Outcome measurements during palliative sedation

Discomfort before and during palliative sedation was monitored with the Discomfort Scale-Dementia of Alzheimer Type (DS-DAT). This observational measurement scale covers nine items for which scores can range from 0 to 3 points, resulting in a total discomfort score ranging from 0 to 27 points. Higher scores represent more discomfort [14, 15]. Scores for the positive items related to content facial expression and relaxed body language were reversed. The DS-DAT was completed after a 5-min period of observation by one of the attending HCPs, 8 h or less before the start, within 6 h after the start of palliative sedation, and then twice daily (morning and evening shift) for the total period during which palliative sedation was administered.

Before starting the observation for the DS-DAT, the same HCP scored sedation levels with the Richmond Agitation-Sedation Scale modified for palliative care inpatients (RASS-PAL). The RASS-PAL is an observational instrument used to assess the severity of agitation and levels of sedation, ranging from + 4 (overtly combative) to − 5 (no arousal by either voice or physical stimulation) [16].

### Statistical analysis

Statistical analysis was performed using SPSS, version 29 (SPSS, 2017, Inc. Chicago, IL), and R software, version 4.2.2 (The R Foundation for Statistical Computing, Vienna, Austria).

Missing data were explored, and when considered missing at random (MAR), they were imputed with multiple imputation [17].

In the descriptive analysis, proportions for categorical variables and means with standard deviations for continuous variables were calculated, and medians with interquartile ranges were calculated for data with skewed distributions.

Linear mixed models were used to estimate the means and changes in repeated measured discomfort levels during palliative sedation. The repeated measurements of the total discomfort score were included as dependent variables, and the “palliative sedation” and “increased duration of palliative sedation” were included as independent variables. A random intercept for participants was included.

Secondary analyses of the discomfort levels included possible confounders or effect-modifying factors within the model, such as age, sex, form of sedation, and setting.

In sensitivity analyses, we analyzed the discomfort measurements of participants who (1) received continuously administered sedation at the time of the measurement and (2) received midazolam as a sedative medication compared to the sample in which other sedative medications were used or added in addition to midazolam.

Individual correlations per participant were calculated between the total scores of the DS-DAT and the RASS-PAL measured at the same time points. All individual correlations were combined within a meta-analysis. Separately, the relation between repeated measurements of the levels of sedation and the discomfort levels was analyzed in a linear mixed model.

Finally, the DS-DAT score was evaluated by analyzing the item total correlations and the internal consistency (Cronbach’s alpha) as measured in the palliative sample in this study.

A detailed description of the study recruitment, population, and other methodological aspects was published previously [18].

## Results

A total of 257 admissions (*n* = 248 participants) were included in the analysis. Nine participants were followed up during two separate admissions. Data sampling ended prematurely for 115 admissions due to discharge to another care setting. Of the remaining participants (*n* = 142), 62 died without and 80 participants with palliative sedation. During the palliative sedation period, discomfort levels were monitored for 78 participants; two of whom were lost to follow-up.

Most participants (*n* = 36, 46%) were admitted to a hospice setting, one third (*n* = 23, 29%) to hospital wards, and the remaining 24% (*n* = 19) to PCUs. Regarding sex, the number of male (*n* = 36, 46%) and female (*n* = 42, 54%) participants was almost equal, and the mean age of the participants was 69 years (SD 12.3). Table 1 shows all baseline characteristics.

**Table 1:** Baseline characteristics (PALSED study), total and per setting

	All *n* = 78	Hospital *n* = 23	PCU *n* = 19	Hospice *n* = 36	*p* value
**Country**					
Belgium	12 (15%)	-	12 (63%)	-	
Germany	7 (9%)	-	7 (37%)	-	
Italy	19 (24%)	-	-	19 (53%)	
Spain	23 (29%)	23 (100%)	-	-	
The Netherlands	17 (22%)	0	-	17 (47%)	
**Gender,** male	36 (46%)	13 (57%)	10 (53%)	13 (36%)	0.2^b^
**Age,** mean (SD)	69 (12.3)	67 (13.1)	72 (9.8)	69 (13.1)	0.5^c^
**Malignant neoplasm of**					
Digestive organs	26 (33%)	9 (39%)	6 (32%)	11 (31%)	0.8^b^
Respiratory organs	12 (15%)	5 (22%)	3 (16%)	4 (11%)	0.5^b^
Breast	10 (13%)	2 (9%)	3 (16%)	5 (14%)	0.8^b^
(Fe)male genital organs	11 (14%)	1 (4%)	3 (16%)	7 (19%)	0.3^b^
Other	19 (24%)	6 (26%)	4 (21%)	9 (25%)	0.9^b^
**Amount of comorbidities**					
0	19 (24%)	7 (30%)	3 (16%)	9 (25%)	0.5^b^
1	22 (28%)	4 (17%)	7 (37%)	11 (31%)	0.3^b^
2	25 (32%)	11 (48%)	7 (37%)	7 (19%)	0.07^b^
3 or more	12 (15%)	1 (4%)	2 (11%)	9 (25%)	0.08^b^
**ECOG PS**^a^**at inclusion**					
2 or lower	8 (10%)	1 (4%)	2 (11%)	5 (14%)	0.2^b^
3	30 (38%)	9 (39%)	8 (42%)	13 (36%)	0.8^b^
4	38 (49%)	11 (48%)	9 (47%)	18 (50%)	1.0^b^
Missing	2 (3%)	2 (9%)	0	0	
**Time (in days) between admission and start PS**, Median [IQR]	14.2 [8.4–31.1]	21.9 [14.7–31.2]	9.4 [6.6–18.2]	13.6 [8.3–33.6]	0.02^a^

*SD *Standard deviation, *ECOG PS *Eastern Cooperative Oncology Group Performance Status, *IQR *Interquartile Range

^a^ECOG PS categorical: grades 0 (fully active) to 4 (completely disabled)

^b^Pearson’s Chi-square test

^c^ANOVA

Table 2 shows that palliative sedation was initiated mostly for two symptoms (*n* = 31, 40%) or for a cluster of three or more symptoms (*n* = 32, 41%). The three most reported primary indications for starting were dyspnea (*n* = 17, 22%), delirium (*n* = 14, 18%), and exhaustion (*n* = 13, 17%). Predominantly, palliative sedation was started directly as continuous sedation (*n* = 73, 94%). In five participants, sedation was intermittently administered, and this modality was used until death in three participants and changed to continuous sedation for two participants. In the total sample, palliative sedation had a median duration of 38 h [IQR 16–88]. In two patients, the dose of medication was reduced until cessation, and the patients died comfortably without the use of sedative medication.

**Table 2:** Palliative sedation characteristics (PALSED study), total and per setting

	All *n* = 78	Hospital *n* = 23	PCU *n* = 19	Hospice *n* = 36	*p* value
**Primary reason to start PS**					
Dyspnea	17 (22%)	7 (30%)	3 (16%)	7 (19%)	0.4^b^
Delirium	14 (18%)	9 (39%)	1 (5%)	4 (11%)	0.01^b^
Exhaustion	13 (17%)	2 (9%)	4 (21%)	7 (19%)	0.5^b^
Existential suffering	9 (12%)	1 (4%)	3 (16%)	5 (14%)	0.4^b^
Anxiety	7 (9%)	0	1 (5%)	6 (17%)	0.07^b^
Pain	5 (6%)	2 (9%)	1 (5%)	2 (6%)	0.9^b^
Nausea/vomiting	4 (5%)	0	3 (16%)	1 (3%)	0.05^b^
Other	7 (9%)	1 (4%)	3 (16%)	3 (8%)	0.5^b^
Missing	2 (3%)	1 (4%)	0	1 (3%)	
**No. of symptoms at start PS**					
1	15 (19%)	8 (35%)	2 (11%)	5 (14%)	0.08^b^
2	31 (40%)	8 (35%)	7 (37%)	16 (44%)	0.7^b^
3 or more	32 (41%)	7 (30%)	10 (53%)	15 (42%)	0.3^b^
**Form of PS**					
Continuous sedation	73 (94%)	22 (96%)	18 (95%)	33 (92%)	0.8^b^
Intermittent sedation	3 (4%)	0	1 (5%)	2 (6%)	0.5^b^
Started intermittent- changed in continuous sedation	2 (3%)	1 (4%)	0	1 (3%)	0.6^b^
**Sedative medication used**					
Midazolam only	50 (64%)	17 (74%)	8 (42%)	25 (69%)	0.07^b^
Midazolam and other medication^c^	26 (33%)	6 (26%)	9 (47%)	11 (31%)	0.3^b^
Midazolam changed in phenobarbital	2 (3%)	0	2 (11%)	0	0.04^b^
**Recalculated midazolam dosages (mg/h),** median [IQR]	2.3 [1.5–3.1]	2.0 [1.2–3.1]	3.1 [2.5–5.1]	2.3 [1.7–2.8]	0.01^a^
**Administration of opioids(sc/iv) during PS, **yes (*n*, %)^d^	74 (95%)	21 (91%)	17 (89%)	36 (100%)	0.2^b^
**Duration PS (in hours), **median [IQR]	38.1 [16.2–87.5]	41.6 [18.6–80.5]	37.0 [27.0–97.8]	31.4 [15.6–72.7]	0.8^a^

*PCU *Palliative Care Unit, *PS *Palliative Sedation, *IQR *Interquartile Range

^a^Kruskal-Wallis

^b^Pearson’s chi-square

^c^Levomepromazine (*n* = 18), Clotiapine (*n* = 7), Ketamine (*n *= 1)

^d^Used opioids: Buprenorphine, Eptadone, Fentanest, Hydromorphon, Morphine, Oxynorm

Midazolam was the first-choice sedative medication for all participants. Midazolam was started and continued at low (< 2 mg/h), moderate (2–5 mg/h), and high dosages (> 5 mg/h) in 31, 25, and 4 of patients, respectively. Adjustments were made to the administered midazolam dosages in 18 participants (23%). In 64% of the sample (*n* = 50), only midazolam was used, with a total median dosage of 2.0 mg/h (IQR 1.2–2.7). In two cases, midazolam was changed to another sedative, while another sedative medication was added to midazolam treatment in 26 participants. In those 28 participants, midazolam was administered with a median dosage of 3.0 mg/h (IQR 2.2–5.0).

For 78 participants who received palliative sedation, a total of 481 discomfort measurements were completed, with a range of 2 to 21 measurements per participant. In 7% (*n* = 32) of the measurements, there were missing data for the complete DS-DAT and RASS-PAL (*n* = 20) or for some missing DS-DAT item scores (*n* = 12). For two participants, the reasons for missing all measurements were loss to follow-up due to an acute start of palliative sedation and discharge to another hospital ward. From the pooled analysis of ten imputed datasets, we concluded that the outcomes shifted by less than 5% compared to the dataset with complete measurements only. Therefore, we used the database with complete measurements (*n* = 449 measurements) for reporting the results.

### Comfort measurements after the start of palliative sedation

**Fig. 1:**
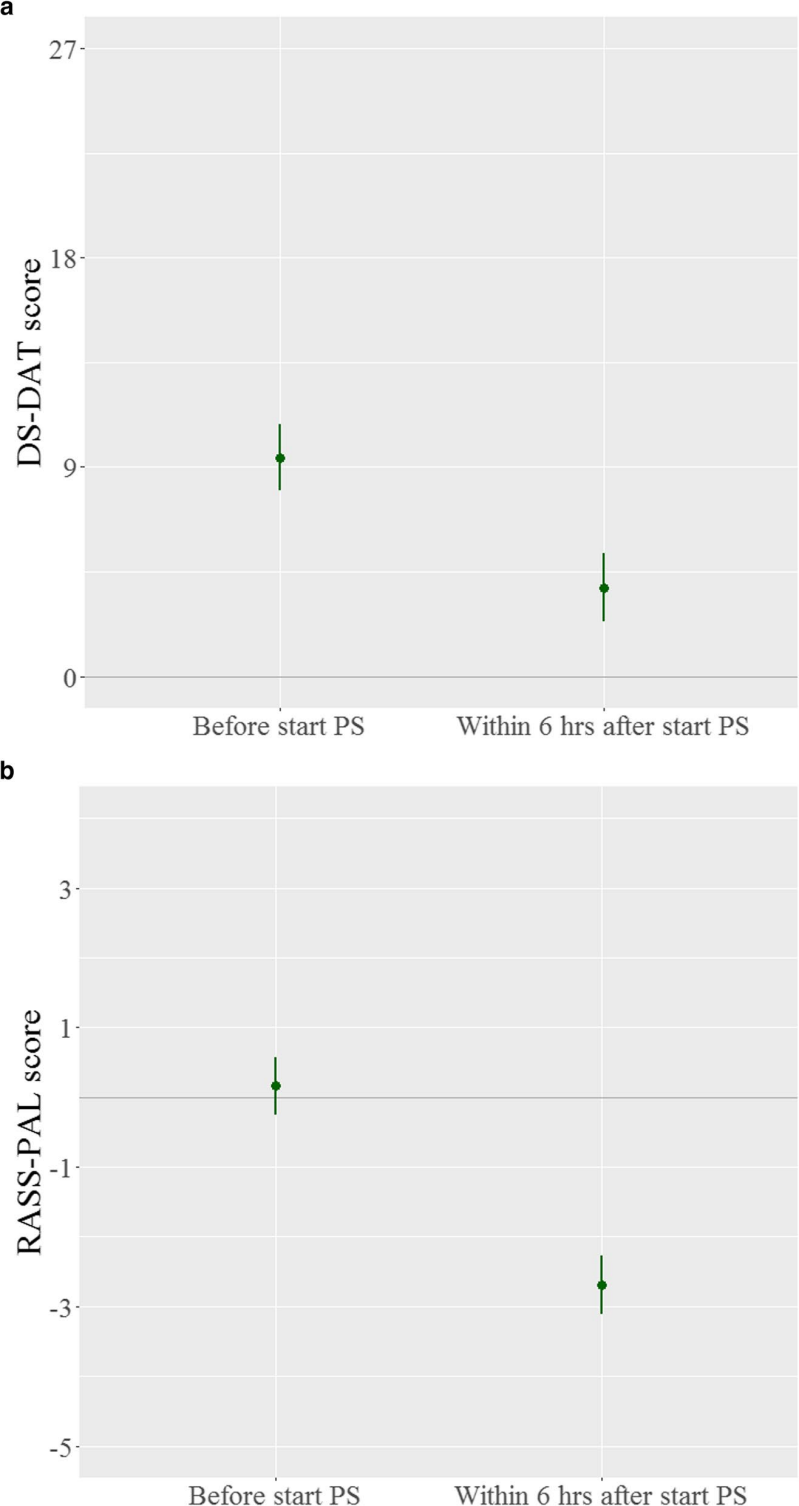
**a** Effect of palliative sedation at discomfort levels (DS-DAT). PS, palliative sedation; DS-DAT, Discomfort Scale-Dementia Alzheimer Type. Measurement before start PS (*n* = 77): mean 9.4 points (95% CI 8.0; 10.9) Measurement within 6 h after start (*n* = 74): mean 3.8 points (95% CI 2.4; 5.3). **b** Effect of palliative sedation at sedation/agitation levels (RASS-PAL). PS, palliative sedation; RASS-PAL, Richmond Agitation-Sedation Scale modified for palliative care. Measurement before start PS (*n* = 77): mean 0.15 points (95% CI -0.27; 0.58); measurement within 6 h after start (*n* = 73): mean -2.69 points (95% CI -3.11; -2.27)

**Fig. 2:**
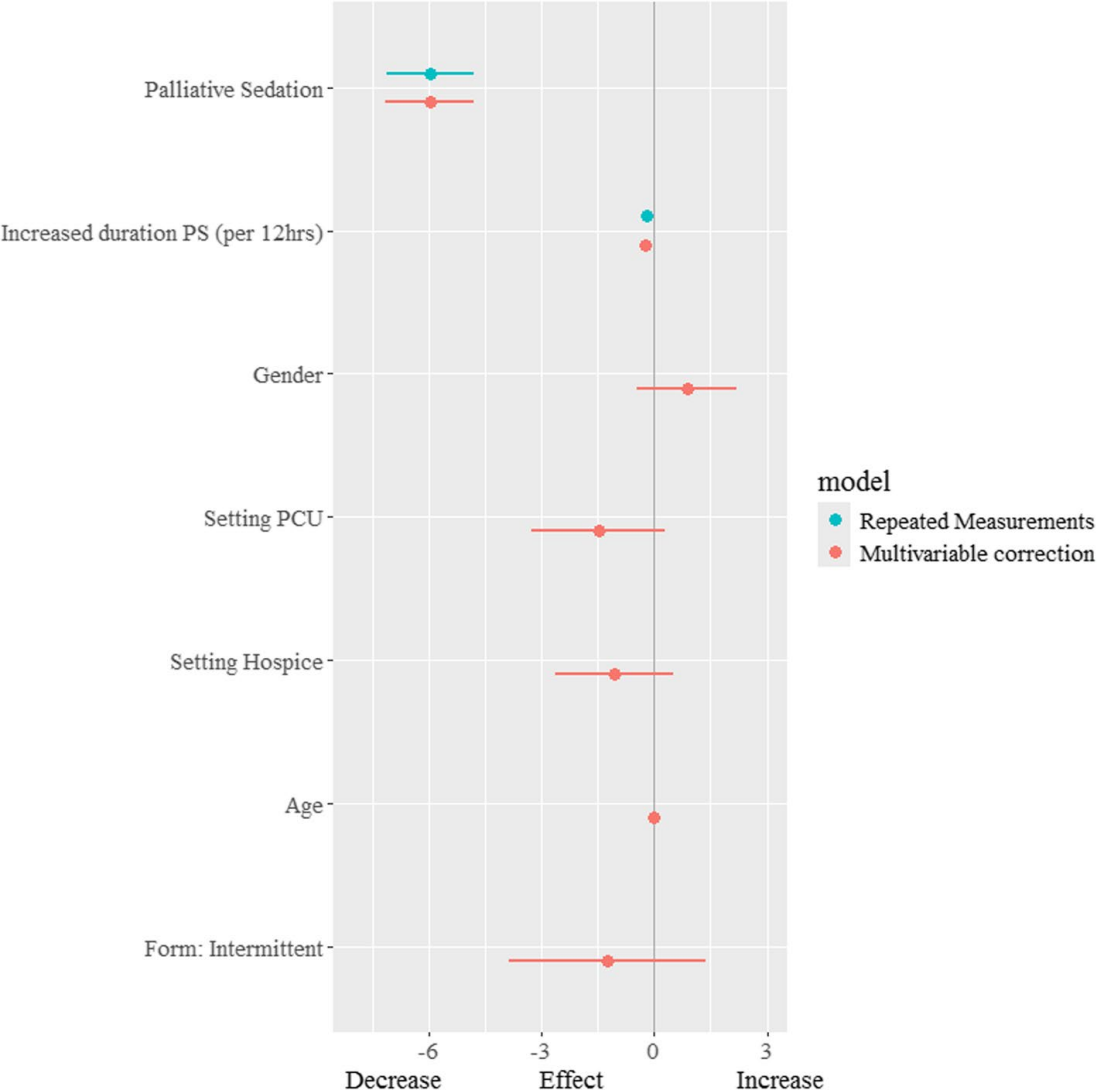
Effects of palliative sedation (PS) and other variables at discomfort levels. PS, palliative sedation; PCU, palliative care unit; 95% CI, 95% confidence interval. Model 1. Repeated measurements model: mean discomfort before start: 9.4 (95% CI 8.3–10.5). Model 2. Multivariable model: repeated measurements model corrected for gender (ref = female), setting PCU and Hospice (ref = Hospital), Age (ref = mean age 69 years), Form: Intermittent sedation (ref = Continuous), mean discomfort before start: 10.0 (95% CI 8.3–11.6)

In the total sample, the mean discomfort score of 9.4 points (95% CI 8.0–10.9) at the start of sedation decreased by 5.6 points (95% CI 3.7–7.5) within 6 h after the start of sedation (Fig. 1a). When all discomfort measurements until the end of the sedation period were used to analyze the effect of palliative sedation, a decrease of 6.0 points (95% CI 4.8–7.1) was shown. Besides the initial effect of palliative sedation on discomfort, each hour increase in the sedation duration also resulted in a small decreasing effect by 0.02 points (95% CI 0.01–0.03) (Fig. 2). Figure 2 shows that in the multivariable analysis (red lines), no significant factors affecting discomfort levels at onset were found. Hospital ward patients had marginally greater mean scores on the DS-DAT (10.0, 95% CI 8.3–11.6), indicating greater discomfort before the start of sedation, while PCU participants seemed to have lower scores (8.6, 95% CI 6.9–10.3). However, the previously demonstrated decrease in discomfort levels remained unchanged after correction for age, sex, modality of sedation, and setting.

### Sensitivity analyses

First, the discomfort measurements (*n* = 421) of the participants (*n* = 75) who received continuous sedation at the time of measurement were evaluated. The results were comparable to those of the complete sample, which included intermittent sedation.

A second sensitivity analysis was performed to compare the outcomes of the midazolam group (*n* = 50) to those of the group in which also other sedative medications were used (*n* = 28). No significant differences in outcomes were found between the two groups. The data are not shown.

### Levels of sedation and the correlation with discomfort levels

The level of agitation/sedation was scored at the same time points as the discomfort measurements. Before the start of sedation, the mean RASS-PAL score was 0.15 points (95% CI -0.270.58), which decreased by 2.9 points (95% CI 2.6–3.3) after the start of palliative sedation, indicating deeper sedation (Fig. 1b).

A deeper sedation score, as measured with the RASS-PAL, correlated with a lower discomfort score (DS-DAT), with an *r* of 0.72 (95% CI 0.61–0.82). This relation was also revealed within a linear mixed model analysis. A decrease of one point in the RASS-PAL score, resulted in a decrease of 1.6 points (95% CI 1.4–1.8) at the DS-DAT.

As a sensitivity analysis, we also analyzed the range of measured discomfort score in participants with different depths of sedation. In participants with drowsy (RASS-PAL-1) to moderate sedation (RASS-PAL-3) measurements (*n* = 144, 32%), the DS-DAT scores ranged from no discomfort (*n* = 59, 41%) to minimal-moderate discomfort (score 1–7, *n* = 61, 42%) to high discomfort (score 8–27, *n* = 24, 17%). Most discomfort measurements in our sample (*n* = 200, 45%) were accompanied by a simultaneously measured deep sedation score. For those participants with RASS scores of -4 or -5, the DS-DAT ranged from no discomfort (*n* = 130, 65%) to minimal-moderate discomfort (score 1–7, *n* = 62, 31%) or high discomfort (score 8–18, *n* = 8, 4%).

### DS-DAT scores in the sample of participants receiving palliative sedation

In the study sample, the internal consistency of the total DS-DAT had a Cronbach’s alpha of 0.83. All corrected item total correlations were > 0.35 (range 0.36–0.68), except for the “Noisy breathing” item, which had a lower correlation value (0.3). The Cronbach’s alpha increased to 0.85 when this item was deleted, while it decreased to less than 0.8 when the “Content facial expression,” “Relaxed body language,” or “Tense body language” items were deleted (see Table 3).

**Table 3:** Reliability statistics of the DS-DAT scale in the study sample^a^

Number of items	Cronbach’s alpha
9	0.82
**Item**	**Cronbach’s alpha if item deleted**
Noisy breathing	0.844
Negative vocalization	0.829
Content facial expression	0.794
Sad facial expression	0.808
Frightened facial expression	0.815
Frown	0.805
Relaxed body language	0.798
Tense body language	0.795
Fidgeting	0.809

*DS-DAT*, Discomfort Scale-Dementia Alzheimer Type

^a^Evaluation scale in 481 measurements of 78 study participants

## Discussion

In this prospective, international, multicenter clinical study, discomfort levels were used to monitor the efficacy of palliative sedation. Discomfort levels significantly decreased in advanced cancer patients from different palliative care settings in which different forms and depths of sedation were used. A continuous decrease in discomfort levels was demonstrated until the last measurement, with the most substantial decrease occurring within the first 6 h after the initiation of palliative sedation.

Similar to the study by van Deijck et al., our analysis of the DS-DAT scores confirmed the presence of discomfort before the initiation of palliative sedation [10]. They reported higher discomfort levels at baseline, possibly due to inclusion of an elderly and broader patient group with various life-limiting diseases in nursing homes and hospice units. Our study focused solely on cancer patients which were also included from hospital wards and PCUs, likely leading to different end-of-life clinical presentations [19–21]. Nevertheless, the mean discomfort score of 9.5 in our study falls within this range as reported by a Dutch validation study of the DS-DAT. Van der Steen et al. concluded, based on their own and previous research, that total scores between 8 and 10 points indicate high levels of discomfort [15].

Baseline discomfort levels decreased significantly within 6 h after the start of palliative sedation. This decrease was for most patients below the cut-off point of 8 without altering initial medication strategies, suggesting a clinically relevant effect [10, 15]. Similar reductions in DS-DAT scores were observed in van Deijck’s study, though the effect size differed slightly (5.4 vs. 4.1) [10]. This might be explained by methodological differences. Unlike our study, they used independent observers which could increase objectivity. Familiarity of the HCPs with patients might have contributed to more nuanced, but potentially less objective evaluations [22].

The levels of agitation/sedation and discomfort were concurrently measured to ensure comparability with previous research. Prior to sedation initiation, the participants were alert and calm according to their RASS-PAL scores (mean 0.2), both in our sample and in Imai’s study [23]. Although we do not know exactly what causes these lower RASS-PAL scores at baseline, one possible explanation could be that this was caused by the lower doses of sedatives and analgesics used for symptom treatment before the start of palliative sedation. However, the baseline DS-DAT score still indicated a degree of discomfort (score of 9.5). After the initiation of palliative sedation, our analysis revealed a decrease in the RASS-PAL score towards moderate sedation and a good correlation between the DS-DAT and RASS-PAL scores, as demonstrated in previous research [23, 24]. The fact that the same healthcare provider measured both the RASS-PAL and the DS-DAT may possibly have influenced the latter observation.

It was previously discussed whether measuring discomfort in the more deeply sedated patients is still informative [22]. Therefore, we found it reassuring to still see some variation in discomfort scores in the patients which had deeper sedation scores.

Considering these points, we support the conclusion of Imai et al. [24] that the RASS-PAL alone might not be specific or sensitive enough for quantifying the efficacy of palliative sedation. To assess whether the observed decrease in consciousness results in adequate symptom control, simultaneously measured discomfort levels add essential information. This is especially the case for patients for whom proportional sedation rather than direct deep sedation is the aim.

Independent of patient characteristics, setting, depth of sedation, and need for sedation adjustments, discomfort levels during palliative sedation could be evaluated with the DS-DAT in our study sample. Internal consistency of the scale in this sample matched that of previous studies [15, 25]. While the DS-DAT provided an overall adequate discomfort assessment, some items were challenging. Difficulties were expressed by the HCPs in distinguishing the “Noisy breathing” from rattling that may be present during the dying process. Whether audible breathing is a sign of discomfort in dying patients is unclear [26]. Lower item-total correlation and improved internal consistency after excluding this item support this notion [15, 23]. Although the items used within the DS-DAT allow for a consistent and nuanced discomfort measure, the detailed and time-intensive nature of the scale limits rapid assessment. A simplified, dichotomous scale with only the most informative items could better support prompt monitoring and treatment adjustments for patients in palliative sedation [27]. Our study and previous studies with the DS-DAT offer a strong basis for future validation within palliative sedated participants, potentially in a modified form.

### Strengths

This is the first European study performed across different countries and palliative care settings in which palliative sedation trajectories were clinically evaluated in a prospective, standardized manner. This study has provided insights into how proxy-measured discomfort levels can contribute to the assessment of the effectiveness of palliative sedation.

The use of a discomfort measurement tool consistent with clinical practice and the use of HCPs from various settings as observers has brought together study participation and attention for the current bedside monitoring process. Thus, the provided feedback and suggestions for improvements came directly from the HCPs themselves. This allows for further research and the implementation of monitoring tools tailored to the demands of clinical practice.

### Limitations

Only 71% (78/110) of the intended sample size was reached due to a decrease in the number of eligible patients during and after the COVID-19 pandemic, likely due a change in place of death [28]. In addition, we faced greater numbers of rapidly deteriorating patients or patients with cognitive impairment who were no longer able to provide informed consent to participate. In future studies, proxy consent might be helpful for increasing the number of included patients.

Although HCPs completed the DS-DAT within the established time intervals, they notably delayed some of the measurements until the patient’s agitation was resolved. Potentially, moments of increased discomfort might have been missed or not measured.

## Conclusions

Palliative sedation is effective in decreasing the discomfort levels of patients with advanced cancer and a clinical indication for palliative sedation. The DS-DAT contributed to the measuring and comparison of changes in discomfort levels during palliative sedation. This adds value to the clinical observations of HCPs in specialized palliative care settings across European countries.

## Data Availability

Data collected for the study and deidentified for all patients will be made available to other researchers. The data includes baseline characteristics such as diagnoses, measurements of symptom burden and performance status, refractory symptoms and indications at start palliative sedation, medication profiles, and discomfort and sedation scores before and during palliative sedation. The study protocol and clinical study report with a summary of study outcomes will also be made available. Data will be made available beginning 9 months following article publication. The data will be available in the Radboud Repository, but without investigator support other than deposited metadata.

## Abbreviations

DS-DAT: Discomfort Scale-Dementia of Alzheimer Type
RASS-PAL: Richmond Agitation-Sedation Scale modified for palliative care inpatients
95% CI: 95% Confidence interval
PCU: Palliative care unit
EAPC: European Association for Palliative Care
HCP: Health care professional
MAR: Missing at random
SD: Standard deviation
IQR: Interquartile range
